# Early Intervention and a Direction of Novel Therapeutics for the Improvement of Functional Outcomes in Schizophrenia: A Selective Review

**DOI:** 10.3389/fpsyt.2018.00039

**Published:** 2018-02-19

**Authors:** Masayoshi Kurachi, Tsutomu Takahashi, Tomiki Sumiyoshi, Takashi Uehara, Michio Suzuki

**Affiliations:** ^1^Arisawabashi Hospital, Toyama, Japan; ^2^Department of Neuropsychiatry, Graduate School of Medicine, University of Toyama, Toyama, Japan; ^3^Department of Clinical Epidemiology, Translational Medical Center, National Center of Neurology and Psychiatry, Tokyo, Japan; ^4^Department of Neuropsychiatry, Kanazawa Medical University, Kanazawa, Japan

**Keywords:** schizophrenia, functional outcome, early intervention, *N*-methyl-d-aspartate receptor, structural MRI

## Abstract

**Background:**

A recent review reported that the median proportion of patients recovering from schizophrenia was 13.5% and that this did not change over time. Various factors including the duration of untreated psychosis, cognitive impairment, negative symptoms, and morphological changes in the brain influence the functional outcome of schizophrenia. The authors herein reviewed morphological changes in the brain of schizophrenia patients, effects of early intervention, and a direction of developing novel therapeutics to achieve significant improvement of the functional outcome.

**Methods:**

A selective review of the literature including studies from our department was performed.

**Results:**

Longitudinal structural neuroimaging studies on schizophrenia revealed that volume reductions in the peri-Sylvian regions (e.g., superior temporal gyrus and insula), which are related to positive psychotic symptoms, progress around the onset (critical stage) of schizophrenia, but become stable in the chronic stage. On the other hand, morphological changes in the fronto-thalamic regions and lateral ventricle, which are related to negative symptoms, neurocognitive dysfunction, and the functional outcome, progress during both the critical and chronic stages. These changes in the peri-Sylvian and fronto-thalamic regions may provide a pathophysiological basis for Crow’s two-syndrome classification. Accumulated evidence from early intervention trials suggests that the transition risk from an at-risk mental state (ARMS) to psychosis is approximately 30%. Differences in the cognitive performance, event-related potentials (e.g., mismatch negativity), and brain morphology have been reported between ARMS subjects who later developed psychosis and those who did not. Whether early intervention for ARMS significantly improves the long-term recovery rate of schizophrenia patients remains unknown. With respect to the development of novel therapeutics, animal models of schizophrenia based on the *N*-methyl-d-aspartate receptor hypofunction hypothesis successfully mimicked behavioral changes associated with cognitive impairments characteristic of the disease. Furthermore, these animal models elicited histological changes in the brain similar to those observed in schizophrenia patients, i.e., decreased numbers of parvalbumin-positive interneurons and dendritic spines of pyramidal neurons in the frontal cortex. Some antioxidant compounds were found to ameliorate these behavioral and histological abnormalities.

**Conclusion:**

Early intervention coupled with novel therapeutics may offer a promising approach for substantial improvement of the functional outcome of schizophrenia patients.

## Introduction

The functional outcome of schizophrenia patients has been a major concern in psychiatry. A systematic review of 50 studies from 1921 to 2011 (1) demonstrated that the median proportion of patients recovering from schizophrenia was 13.5% and that this did not change over time despite the progress in treatment in recent decades.

Various factors have been reported to influence the course and outcome of schizophrenia patients. The predictors of the 2-year outcome in the WHO 10-country study (2) were the age, sex, marital status, mode of onset (acute or insidious), duration of untreated psychosis (DUP), premorbid psychosocial functioning, close friends, drug abuse, and sociocultural setting, i.e., a developing versus a developed country. However, most of these factors except DUP and drug abuse are beyond clinical control.

Other important factors include neurocognitive dysfunctions (3–6), negative symptoms (7, 8), and alterations in brain morphology (9–11). Although antipsychotic medications are effective for reducing positive psychotic symptoms, these symptoms including first-rank symptoms showed no significant correlation with the outcome of schizophrenia patients (2, 3). The improvements of cognitive impairment and negative symptoms are not satisfactory with the current treatment, and they may have pathophysiologies that differ from those of positive symptoms. It should be noted that there is compelling evidence that the progressive enlargement of the lateral ventricles is closely related to the outcome of schizophrenia patients (9–11). These morphological brain changes should be the target of novel therapeutics.

Duration of untreated psychosis, neurocognitive dysfunctions, negative symptoms, and brain morphology are potentially controllable by medicine. An effective method to substantially improve the functional outcome would be to develop a therapeutic strategy to control these factors. Therefore, the authors reviewed studies on morphological changes in the brain of schizophrenia patients, early intervention, and a direction of developing novel therapeutics.

## Methods

The method was a selective review of the literature including studies from the authors’ department. Concerning studies on morphological brain changes associated with functional outcome in schizophrenia patients, the authors searched PubMed using the keywords: “MRI,” “functional outcome,” and “schizophrenia.” Combination with the author’s previous manual list resulted in 15 publications relevant to the present theme as shown in Table 1. Some of these studies were the starting point of this review. In the section of novel therapeutics, potential candidate compounds are described, capable of ameliorating the histological brain changes in schizophrenia patients.

**Table 1:** Morphological changes in the brain related to the functional outcome in schizophrenia patients.

Study	Year	Subjects	Methods (intervals between scans)	Findings
Davis et al. (9).	1998	53 chronic patients (22 Kraepelinian and 31 non-Kraepelinian)	Longitudinal CT (mean intervals of 5 years)	The ventricles showed a bilateral increase in size over 4-year interval in the Kraepelinian subgroup, more marked in the left hemisphere than the right
Lieberman et al. (10)	2001	51 first-episode patients and 13 controls	Longitudinal MRI (at least 12 months)	Progressive ventricular enlargement in patients with poor outcome schizophrenia
Ho et al. (12)	2003	73 recent-onset patients and 23 controls	Longitudinal MRI (mean intervals of 3 years)	Patients with poor outcome had a greater lateral ventricular enlargement over time than patients with good outcome
Brickman et al. (13)	2004	106 chronic patients and 42 controls	MRI	Patients showed significantly smaller thalamic areas, and the effects were most marked in the patients with poor outcome
Mitelman et al. (14)	2005	37 chronic patients and 37 controls	MRI	Poor outcome subgroup exhibited significant bilateral gray matter deficits in posterior cingulate and retrosplenial cortices compared to good outcome patients
Cahn et al. (15)	2006	31 first-episode patients	Longitudinal MRI (1 year) and 5-year outcome	Progressive brain volume changes of gray matter during the first year of illness were significantly associated with clinical and functional outcome 5 years after the first episode
Mitelman et al. (16)	2006	104 chronic patients (51 good outcome and 53 poor outcome) and 41 controls	Diffusion tensor imaging	Overall white matter fractional anisotropy was reduced in patients with poor outcomes in both hemispheres
Wood et al. (17)	2006	46 patients with first-episode psychosis	Proton MRS	Low scores on the NAA/Cr ratio in the prefrontal cortex were related to poorer outcome
van Haren et al. (11)	2008	96 patients and 113 controls	Longitudinal MRI (over 5 years)	Poor outcome patients showed more brain tissue loss during the follow-up interval than good outcome patients
Wobrock et al. (18)	2009	45 first-episode patients	MRI, follow-up to 1 year	A significant reduced area of the left anterior limb of the internal capsule in patients with clinically relevant deterioration compared to those with stable psychopathology
Mitelman et al. (19)	2009	Chronic schizophrenia (26 poor outcome and 23 good outcome patients) and 16 controls	Longitudinal MRI (4 years)	The rate of decline in volumes of the putamen was greater in patients with poor outcome than in the good outcome group
Mitelman et al. (20)	2010	49 chronic patients and 16 controls	Longitudinal MRI (4 years)	Progressive enlargement of the posterior horn in the poor outcome (Kraepelinian) group
van Haren et al. (21)	2011	96 patients and 113 controls	Longitudinal MRI, vertex-by-vertex basis (5 years)	Frontal and temporal cortices showed excessive thinning over time, possibly related to outcome and medication intake
Tully et al. (22)	2014	26 patients and 29 controls	MRI, surface-based morphometry	Cognitive control fully mediated the relationship between cortical thickness in the superior frontal gyrus and role functioning
Dusi et al. (23)	2017	Chronic schizophrenia (35 poor outcome, 35 good outcome patients, and 76 controls)	Longitudinal MRI (3 years)	At baseline, poor outcome patients showed significantly decreased right dorsolateral prefrontal cortex (DLPFC) white matter volumes compared to controls, with shrinkage of left DLPFC white matter volumes at follow-up

## Structural Magnetic Resonance Imaging Studies on Schizophrenia

### Region of Interest Method

Structural magnetic resonance imaging (sMRI) studies using the region of interest method have demonstrated significant volume reductions in schizophrenia patients mainly in three brain regions: the medial temporal lobe structures (hippocampus and amygdala), peri-Sylvian regions (superior temporal gyrus and insula), and prefrontal areas including fronto-thalamic connections, compared with healthy control subjects. We noted the difference in brain morphology between patients with schizophrenia and subjects with schizotypal (personality) disorder or first-degree relatives of the patients to differentiate the disease process from risk- or vulnerability-associated changes.

Among the three regions, volume reductions in the medial temporal lobe structures have been confirmed in schizophrenia patients (24). However, these changes may represent a risk of or vulnerability to the disease, as pointed out by Seidman et al. (25), since these changes were also seen to the same extent in schizotypal (personality) disorder and first-degree relatives of patients with schizophrenia (25–27). A meta-analysis of longitudinal sMRI studies on schizophrenia (28) showed no evidence to suggest progressive medial temporal lobe involvement.

Volume reductions in the superior temporal gyrus were seen in both schizophrenia and schizotypal disorder patients (29, 30). However, the changes in schizophrenia patients were more widespread than in schizotypal disorder patients: the changes extended to Heshcl’s gyrus and the planum polare in schizophrenia, but not in schizotypal disorder (30). In longitudinal studies, progressive decreases in the gray matter volume in the left superior temporal gyrus were noted in first-episode schizophrenia patients (31, 32). During the chronic stage, however, no significant progressive changes of the superior temporal gyrus were seen in schizophrenia patients using the region of interest method (33, 34).

Volume reduction in the insular cortex was noted in schizophrenia patients but not in schizotypal patients compared with healthy control subjects (35, 36). On longitudinal comparison, first-episode patients showed a significant gray matter reduction of the insular cortex over time compared with controls (37, 38). In chronic schizophrenia patients, however, no significant changes were found (38).

Thus, volume reductions in the superior temporal gyrus and insular cortex have been shown to progress during the first episode, but they may become stable in the chronic stage.

With regard to the prefrontal areas, schizophrenia patients showed reductions in the volume of the anterior cingulate gyrus, dorsolateral prefrontal areas (superior, middle, and inferior frontal gyri), and straight gyrus, while schizotypal patients had larger volumes of the bilateral middle frontal gyrus (26) or Brodmann area 10 (39) compared with healthy controls. Yamasue et al. (40) demonstrated that the effect size of volume reductions in schizophrenia patients was largest in the anterior cingulate gyrus among prefrontal and temporolimbic regions (superior temporal gyrus, amygdale–hippocampus complex, insula, and anterior cingulate gyrus).

In longitudinal studies, early schizophrenia patients showed a progressive reduction in the frontal lobe white matter volume, which was associated with more negative symptoms (12). A meta-analysis of 27 longitudinal volumetric studies (28) revealed that patients with schizophrenia showed significantly greater decreases over time in the frontal gray and white matter, parietal white matter, and temporal white matter volumes than healthy controls. Thus, frontal lobe alterations in schizophrenia may progress in patients, at least in part, through the first episode and chronic stage.

Slight but significant enlargement of the lateral ventricle, especially in the left hemisphere, is one of the most consistent morphological brain changes in schizophrenia patients (41, 42). This enlargement is most likely due to the volume reductions of the adjacent white matter of the brain. With regard to this, Suzuki et al. (43) and Zhou et al. (44) reported white matter reductions in the anterior limb of the internal capsule in schizophrenia patients. It is interesting to note that bidirectional glutamatergic fronto-thalamic fibers pass through the anterior limb of the internal capsule. In longitudinal studies on first-episode schizophrenia, it was found that poor outcome patients showed an increase in the ventricle volume over time, whereas the ventricle volume of good outcome patients and controls did not change (10). In chronic schizophrenia, progressive ventricle enlargement has also been reported in poor outcome patients (9, 11).

### Voxel-Based Morphometry (VBM)

The VBM method has the advantage of being able to explore local morphological changes in the whole brain. Suzuki et al. (43) using voxel-based analysis, reported that the gray matter in schizophrenia patients was significantly reduced in the medial temporal, left superior temporal, left middle and inferior frontal, right inferior frontal, and bilateral anterior cingulate areas compared with healthy controls.

Kawasaki et al. (45) studied gray matter changes in schizophrenia and schizotypal disorder patients compared with healthy controls by VBM of three-dimensional MRI. They found that the volume of the medial temporal lobe structure and superior temporal gyrus was reduced in both schizophrenia and schizotypal disorder patients compared with healthy controls, whereas volume reductions in the frontal gyri were prominent only in schizophrenia patients.

Subsequent meta-analyses of VBM studies (46, 47) revealed gray matter reduction in a network of frontal, temporal, thalamic, and striatal regions in schizophrenia patients relative to healthy comparison subjects. Despite some discrepancies (48), these results are consistent with those using the region of interest method and support the view of Siever and Davis (49) and Kurachi (50) that frontal lobe alterations play a crucial role in the development of schizophrenia.

Honea et al. (51), using the VBM method, demonstrated that schizophrenia patients showed volume reductions in the bilateral superior and middle frontal gyri, left inferior frontal gyrus, and right thalamus compared with their unaffected siblings with no history. Furthermore, Hao et al. (52), using voxel-based analysis of diffusion tensor imaging, demonstrated that both schizophrenia patients and their healthy siblings showed reduced white matter fractional anisotropy in the left prefrontal cortex and hippocampus in comparison with healthy controls, while only schizophrenia patients exhibited reduced white matter fractional anisotropy in the left anterior cingulate cortex in comparison with both siblings and controls.

Thus, cross-sectional region of interest as well as VBM sMRI studies showed volume reductions in medial temporal lobe structures, peri-Sylvian regions, and prefrontal areas including fronto-thalamic connections in schizophrenia patients compared with healthy controls. Longitudinally, volume reduction in the peri-Sylvian regions (e.g., superior temporal gyrus and insula) progressed during the first episode, but became stable in the chronic stage. On the other hand, morphological changes in the frontal lobe and lateral ventricle progressed through the first episode and chronic stage.

## Clinical Correlates of Morphological Changes in Schizophrenia and a Pathophysiological Model

Several studies have reported clinical correlates of morphological changes in schizophrenia patients. Auditory hallucinations have been reported to be associated with gray matter volume reductions in the left (anterior) superior temporal gyrus and Heschl’s gyri by the region of interest method (30, 53) and a subsequent VBM study (54).

Shenton et al. (55) reported that the severity of formal thought disorder was negatively correlated with the volume of the left posterior superior temporal gyrus, and a VBM study (56) also showed the correlation with the gray matter volume reduction within the left temporal lobe in addition to the right middle orbital and cuneus/lingual gyri.

According to the study by Takahashi et al. (38), the gray matter loss of the left insular cortex over time in first-episode patients was correlated with the severity of positive and negative symptoms on follow-up.

Thus, the structural alterations in the peri-Sylvian regions (e.g., superior temporal gyrus and insula) were related to positive psychotic symptoms such as auditory hallucinations, thought disorder, and possibly delusions.

With regard to negative symptoms, a correlation with functional and structural alterations in prefrontal areas and the medial thalamus has been reported (12, 57). This correlation was clearly evidenced by a VBM study (58) although insular gray matter volume may also be involved in negative symptoms (59).

In addition to positive and negative symptoms, neurocognitive dysfunctions, especially executive dysfunctions, are an important aspect of schizophrenia, since they are related to the functional outcome (3). A meta-analysis of 41 functional neuroimaging studies of the executive function in schizophrenia patients (60) revealed that patients with schizophrenia showed reduced activity in the dorsolateral prefrontal cortex, anterior cingulate cortex, and mediodorsal nucleus of the thalamus. Consistent with this, a sMRI study showed that the executive dysfunctions were correlated with volume reduction in the bilateral dorsolateral prefrontal cortex in schizophrenia patients (61). Regarding memory impairment, deficits in memory organization have been shown to be a characteristic feature in schizophrenia patients (62). Memory organization deficits were related to volume reduction in the prefrontal cortex (63).

Studies on the morphological brain changes related to the functional outcome in schizophrenia patients are summarized in Table 1. First, in accordance with the report by Keefe et al. (64), which suggested morphological brain changes, e.g., ventricle enlargement, in the Kraepelinian (most severely deteriorated) chronic schizophrenic patients, progressive enlargement of the lateral ventricle has been one of the most consistent findings in poor outcome patients (9–12). This enlargement is at least in part derived from the volume reduction of the anterior limb of the internal capsule, through which the bidirectional fronto-thalamic fibers pass. Volume reductions in the anterior limb of the internal capsule in schizophrenia patients were related to verbal and spatial memory (65) and the social outcome (18). Furthermore, a marked reduction in sleep spindles in schizophrenia patients also suggests impairment of the thalamo-frontal circuitry in this disease (66–68).

Second, prefrontal alterations were reported to be related to a poor outcome in schizophrenia patients (17, 22, 23). Notably, Tully et al. (22) reported that the relationship between the prefrontal alterations and poor outcome were mediated by impaired cognitive control (category fluency).

Third, other brain areas were also mentioned (11, 16, 21). van Haren et al. (21) reported progressive decreases in the cortical thickness in the superior temporal cortex as well as anterior cingulate cortex in patients with poor outcomes based on the vertex-by-vertex method. These findings appear to disagree with the results using the region of interest method (33, 34). In addition, the study by van Haren et al. is characterized by a large sample with a wide ranging duration of illness.

With regard to outcome measures, clinical criteria by Keefe et al. (64) have been frequently used as well as the Global Assessment of Functioning (GAF) score. In 2013, the American Psychiatric Association (69), instead of the GAF, adopted the WHO Disability Assessment Schedule (WHODAS 2.0) in Diagnostic and Statistical Manual of Mental Disorders-5.

To briefly summarize, volume reductions in the peri-Sylvian regions are mainly related to positive symptoms, whereas alterations in the regions composing the fronto-thalamic circuitry are mainly related to negative symptoms, executive functions, and the functional outcome. These two types may be called the peri-Sylvian type and fronto-thalamic type, respectively, and this model may provide a pathophysiological basis for Crow’s (70) positive and negative syndrome classification (Table 2). Traditional simple type schizophrenia is a good example of the fronto-thalamic type, as evidenced by Suzuki et al. (71). This model could explain why positive psychotic symptoms are not significantly correlated with the functional outcome, since it proposes that positive psychotic symptoms and the functional outcome have distinct underlying pathophysiologies.

**Table 2:** Pathophysiological modeling of schizophrenia.

Type	Peri-Sylvian type	Fronto-thalamic type
Brain regions involved	Superior temporal gyrus, insula	Anterior cingulate gyrus
		Dorsolateral prefrontal gyrus
		Dorsomedial thalamus

Chemical pathology	Excessive DA neurotransmission	Imbalance in glutamate-GABA system

Clinical manifestations	Positive symptoms (delusions, hallucinations, disorganized speech)	Negative symptoms (diminished emotional expression, avolition)
		Executive dysfunctions
		Disability in social functioning

Clinical course	Progressive during the prodromal and first-episode stages, but stable in the chronic stage	Progressive both in the first episode and chronic stages

Responsiveness to treatment	Responsive	Refractory

*DA, dopamine; GABA, gamma-aminobutyric acid*.

From the viewpoint of this pathophysiological model, it is noteworthy that the morphological brain changes in schizophrenia patients of discordant and concordant twins are not identical. According to a VBM study (72), patients with schizophrenia of discordant twins showed volume reductions in peri-Sylvian regions as well as the fronto-thalamic circuitry, whereas patients with schizophrenia of concordant twins showed volume reductions in the fronto-thalamic circuitry, but not in the peri-Sylvian regions.

## Early Intervention

Early intervention consists of two parts: one is early intervention in first-episode psychosis, and the other is that toward the prodromal phase. The main aim of early intervention in first-episode psychosis is to reduce the DUP. Hegelstad et al. (73) studied the effect of this reduction on the 10-year outcome. As a result, 30.7% of the patients from the early-detection area (a median DUP of 5 weeks) fulfilled recovery criteria, while only 15.1% of the patients from the usual-detection area (a median DUP of 16 weeks) did so. For the further improvement of the recovery rate, early intervention toward the prodromal phase would be required.

Since a prodrome is a retrospective concept, the term of “at-risk mental state (ARMS)” has been used in prospective trials (74). Early intervention for help-seeking individuals with ARMS will be necessary, since such subjects suffer the distress of symptoms and are liable to develop disability in functioning.

Importantly, in longitudinal studies, volume reductions in the planum temporale, caudal superior temporal gyrus, and insular cortex progressed in ultra-high-risk (UHR) subjects who later developed psychosis compared with controls or UHR subjects who did not develop it (32, 75). Hence, volume reductions in the peri-Sylvian regions (e.g., superior temporal gyrus and insula) have been shown to progress during the prodromal phase.

The transition risk from ARMS to psychosis was 29–36% over a 2- to 3-year follow-up (76). This figure is markedly higher than the incidence rate of psychosis in the general population, but it should be noted that two-thirds of subjects were false positive using these ARMS criteria.

Thus, for an indicated early intervention, it is necessary to diagnose those who are truly in the prodromal phase among ARMS patients. Effective examination would detect the subclinical pathophysiological process. In accordance with this, several candidate biomarkers have been reported, such as the neurocognitive function, event-related potential, and sMRI. In addition, the Minnesota Multiphasic Personality Inventory may be a useful tool to assess the risk of transition to psychosis.

The cognitive function is impaired in ARMS patients as well as in first-episode and chronic schizophrenia patients (6). In particular, the verbal memory and executive functions of ARMS subjects who later developed psychosis were reported to be lower than in those who did not develop psychosis (77–79). Notably, ARMS patients who developed psychosis or did not show remission during the 2-year follow-up showed a similar impairment in the global cognitive function at the baseline to that in first-episode psychosis patients (79). In addition, the disease transition was predicted by multivariate pattern recognition of the neurocognitive performance (78).

Mismatch negativity (MNN) is a component of event-related potentials that reflects preattentive auditory sensory memory. MMN amplitudes are likely to provide an index of *N*-methyl-d-aspartate (NMDA) receptor-mediated neurotransmissions (80–82). It has been reported that converters to psychosis elicit a reduced amplitude of duration MMN (dMMN), i.e., MMN in response to duration deviants, relative to non-converters (83–85). Hence, it may be possible to use dMMN to predict the conversion from ARMS to psychosis (86, 87).

Concerning the morphological changes in the brain, Koutsouleris et al. (88) revealed that ARMS patients with subsequent disease transition showed prefrontal alterations relative to those in ARMS patients without subsequent disease transition and healthy controls. Subsequently, Koutsouleris et al. (78) showed that the early prediction of psychosis may be reliably enhanced using neuroanatomical pattern recognition at the single-subject level.

A systematic review and meta-analysis (89) demonstrated decreased prefrontal, cingulate, insular, and cerebellar gray matter volumes in high-risk subjects with subsequent transition to psychosis compared with high-risk subjects without transition. In particular, the thickness of the anterior cingulate gyrus was significantly reduced in individuals with ARMS who later developed psychosis relative to healthy subjects (47, 90, 91). These findings, together with those by Yamasue et al. (40) and Hao et al. (52), described in the previous section, suggest the crucial role of the anterior cingulate gyrus in the emergence of schizophrenic symptoms, in which self-disturbance might be fundamental (92, 93).

With regard to the dorsolateral frontal cortex, Reniers et al. (94) reported that lower baseline gray matter densities in the middle and inferior frontal gyri were significantly correlated with a decline in the GAF score over the follow-up, regardless of the transition status or persistence of ARMS. These findings along with the Kopelowicz et al.’s report (5) that frontal lobe functioning (executive function, verbal fluency, and verbal working memory) was associated with recovery from schizophrenia suggest the significance of the prefrontal lobe in social functioning and recovery from this condition.

Minnesota Multiphasic Personality Inventory, consisting of 550 questionnaires, is an established tool to assess personality and psychopathology (95). In our experience in the early intervention project, high scores on Scale 8 (schizophrenia) were associated with subsequent transition to psychosis (96). Subtle alterations of subjective experience may precede changes in objective measures as stated by Klosterkötter et al. (97) and Parnas and Handest (98).

Concerning the early intervention trials including antipsychotic medication or psychological intervention for ARMS patients, a meta-analysis of randomized controlled trials revealed that the overall risk of transition to psychosis was reduced by 54% at the 12-month follow-up (99). In view of the report that cognitive remediation improved memory and psychosocial functioning in first-episode psychiatric outpatients (100) and functional connectivity in early-course schizophrenia patients (101), cognitive remediation may also be effective for ARMS patients.

Whether early intervention in the prodromal phase significantly improves the long-term recovery rate of schizophrenia patients remains elusive. To answer this question, follow-up studies of ARMS patients who subsequently developed psychosis are needed.

In the next section, potential candidate compounds are described, capable of ameliorating the subclinical pathophysiological process, particularly, the histological brain changes in schizophrenia patients.

## A Direction of Novel Therapeutics

The understanding of the disease is composed of three levels: symptomatic, pathophysiological, and etiological. Therapeutics have been developed corresponding to these three levels. Current pharmacotherapy for schizophrenia remains at the symptomatic level, and so the long-term recovery rate has not changed, as described in the previous session. Owing to the recent development of technologies in neuroscience, our understanding of the pathophysiological disease process of schizophrenia has markedly progressed.

Olney and Farber (102) proposed that NMDA receptor hypofunction was a key mechanism that can help explain major clinical and pathophysiological aspects of schizophrenia, including the occurrence of structural brain changes, and stated that NMDA receptor hypofunction on GABAegic neurons would reduce inhibitory control over multiple downstream neurons.

Garey et al. (103). and Glantz and Lewis (104) reported a reduced dendritic spine density on pyramidal neurons in layer of the prefrontal and temporal cortex in postmortem brains of schizophrenia patients, and this was considered to explain the loss of cortical volume without the loss of neurons under this condition.

Furthermore, Reynolds et al. (105) and Zhang and Reynolds (106) reported a loss of parvalbumin–immunoreactive interneurons in the dorsolateral prefrontal cortex and hippocampus in schizophrenia patients. Chung et al. (107) demonstrated that the excitatory synapse density is selectively lower on parvalbumin interneurons in schizophrenia patients and that this may lead to the alterations of cortical gamma oscillations and working memory dysfunction.

Thus, a reduced dendritic spine density on pyramidal neurons and a loss of parvalbumin–immunoreactive interneurons in the cerebral cortex may be core features of histological changes in the brain of schizophrenia patients.

Importantly, animal models of schizophrenia, constructed on the basis of the NMDA receptor hypofunction hypothesis, successfully mimic these histological changes in the brain of schizophrenia.

Nakatani-Pawlak et al. (108) reported that mice neonatally treated with phencyclidine showed impairments of spatial working memory and social interaction behavior in adulthood, in addition to decreases in the number of parvalbumin-positive cells and spine density in the frontal cortex, nucleus accumbens, and hippocampus. Uehara et al. (109, 110) also reported augmented MAP-induced hyperlocomotion, sensorimotor gating deficits, and a loss of GABAergic parvalbumin-positive neurons in rats neonatally exposed to MK-801, an antagonist of the NMDA receptor.

Thus, utilizing these rodent animal models, it became possible to explore or develop novel therapeutics to improve cognitive deficits and the histological changes in the brain of schizophrenia patients.

There may be at least two approaches to develop novel therapeutics. One is to stimulate the glycine/d-serine modulatory site on the NMDA receptor with glycine (111) or d-serine (112). Another strategy is to explore medicines that ameliorate dysfunctional GABAergic neurons. The latter strategy is based on the concept that the hypofunction of NMDA receptors located on GABAergic neurons leads to the attenuated activity of GABAergic neurons, and this, in turn, produces abnormal gamma oscillations and cognitive deficits in schizophrenia patients (113).

Based on these lines, several candidate compounds have been reported. First, the ketamine-induced loss of parvalbumin-positive interneurons has been reported by an increase in brain superoxide due to the activation of NADPH oxidase in neurons (114). Subsequently, Zhang et al. (115) reported that apocynin, an inhibitor of NADPH oxidase, attenuated the cognitive impairments and downregulation of parvalbumin and glutamic acid decarboxylase 67 in rats after repeated ketamine exposure during the neonatal period.

Second, Shirai et al. (116) reported that the antioxidant sulforaphane, found in cruciferous vegetables, significantly attenuated hyperlocomotion and the prepulse inhibition deficits in mice after phencyclidine administration. Furthermore, the dietary intake of sulforaphane-rich broccoli sprout extracts attenuated cognitive deficits and the decrease in parvalbumin-positive cells in the medial prefrontal cortex and hippocampus of these mice (117).

Third, Uehara et al. (110, 118) found that T-817MA, a novel neurotrophic agent, restores parvalbumin-positive GABAergic neurons in the prefrontal cortex and hippocampus of the rat models described above. Haloperidol and risperidone showed no such effect. T-817MA is a newly synthesized agent that was developed for the treatment of neurodegenerative disorders, such as Alzheimer’s disease, and it is markedly protective against Aβ-induced or H_2_O_2_-induced neuronal death (119).

Furthermore, Nakamura et al. (120) reported that the oral administration of T-817MA ameliorated behavioral, histological, and neurophysiological changes, such as deficits in prepulse inhibition, reduced levels of parvalbumin-immunoreactive neurons in the medial prefrontal cortex, hippocampus, and amygdala, and a deficit in the auditory phase-locked gamma oscillation in a mouse model of schizophrenia. The modulation of gamma band activity is noteworthy, because abnormal gamma band activity is thought to underlie the psychosis and cognitive deficits, and is considered a target for potential therapeutic interventions (113).

These compounds have antioxidant effects in common (114, 116, 119). In accordance with this, the antioxidant *N*-acetyl cysteine was reported to prevent the reduction of prefrontal parvalbumin interneuron activity as well as electrophysiological and behavioral deficits in the animal models of schizophrenia (121).

Considering that the dysfunction of parvalbumin-positive GABAergic neurons by NMDA receptor antagonists was mediated by oxidative mechanisms (114), some antioxidants might be novel therapeutics or lead compounds to ameliorate the cognitive deficits and histological disease process in schizophrenia patients.

## Conclusion

Structural neuroimaging studies on schizophrenia revealed that volume reductions in the peri-Sylvian regions are mainly related to positive symptoms, whereas alterations in the fronto-thalamic regions are mainly related to negative symptoms, executive functions, and the functional outcome. These two types, i.e., the peri-Sylvian type and fronto-thalamic type, may provide a pathophysiological basis for Crow’s (70) positive and negative syndrome classification. This model may explain why positive psychotic symptoms are not significantly correlated with the functional outcome, since it proposes that positive psychotic symptoms and functional outcomes are associated with distinct pathophysiology.

Accumulated evidence from early intervention trials suggests that the transition rate to psychosis is approximately 30% among individuals with ARMS. Differences in the cognitive performance, MNN, and brain morphology have been reported between ARMS patients who later develop psychosis and those who do not. The prefrontal lobe function may have a significant role in social functioning and recovery from schizophrenia. Whether early intervention for ARMS significantly improves the long-term recovery rate of schizophrenia patients remains elusive.

With respect to the development of novel therapeutics, animal models of schizophrenia based on the *N*-methyl-d-aspartate receptor hypofunction hypothesis showed histological changes in the brain that successfully mimicked those in the postmortem brains of schizophrenia patients, i.e., decreased numbers of parvalbumin-positive interneurons and dendritic spines of pyramidal neurons in the frontal cortex, in addition to behavioral abnormalities associated with cognitive impairment. Some antioxidant compounds, e.g., apocynin, sulforaphane, and T-817MA, have been found to ameliorate histological changes in the brain and cognitive dysfunction in these animal models.

In conclusion, early intervention coupled with novel therapeutics, herein reviewed, may provide a promising strategy to substantially improve the functional outcome of schizophrenia patients. However, further studies are needed to evaluate the functional outcome in relation to these therapeutic strategies, which is beyond the scope of this review.

## Author Contributions

MK wrote the first draft of the manuscript. TT, TS, TU, and MS contributed to the cited studies and discussed the content of this manuscript.

## Conflict of Interest Statement

The authors declare that the research was conducted in the absence of any commercial or financial relationships that could be construed as a potential conflict of interest.
